# The Management of Chronic Aural Suppuration

**Published:** 1909-03-20

**Authors:** 


### Otology.

### THE MANAGEMENT OF CHRONIC AURAL SUPPURATION.

### II.—OPERATIVE TREATMENT.

In the previous article we considered the disinfection of the ear with lotions, drops, and insufflations; but it is obviously essential first of all to ensure adequate drainage. When the perforation in the drum is very small, and especially when it is situated high up on the membrane, syringing the external meatus washes away only the overflow of discharge and cannot reach the accumulated pus within the ear. It is then necessary to enlarge the opening by incision of the drum; or, better, to clip away a little of the membrane with tiny punch-forceps, for a simple incision tends to close too rapidly. In cases where the perforation is slightly larger, the surgeon may pass an intra-tympanic cannula through the opening and syringe away the discharge; and in some cases antiseptic drops may be made to penetrate by pouring them into the meatus with the head inclined to the sound side, and then inflating the ear by Politzer's or Valsalva's method. When the perforation is large, but the discharge is viscid or semi-solid, this may be evacuated by syringing or inflating through a catheter passed into the Eustachian tube.

A frequent cause of imperfect drainage is the presence of polypi or sprouting granulations; these must be treated, according to their size, by the electric cautery, by chromic or trichloracetic acid, or by removal with curette or snare. It must always be remembered that aural polypus is not a disease, but only a symptom of disease. Occasionally in chronic aural suppuration an accumulation of epithelium takes place from excessive desquamation of the cells of the mucous membrane lining the cavities. This forms a mass known as a "cholesteatoma," and is probably due to ingrowth of the squamous epithelium from the margins of the perforation in the drum membrane; the exciting cause appears to be a chronic inflammation of the underlying bone. As the result of the pressure of the mass, absorption of the bone occurs, and a cavity of large size may be formed, so that even the dura mater and lateral sinus may be exposed. These cholesteatomatous masses must be thoroughly removed and their presence is in general an indication for the radical mastoid operation.

Caries of the ossicles or of the bony walls is an important cause of the continuance of suppuration. Of the ossicles the incus is the most, and the stapes the least, frequently affected. This caries of the ossicles often occurs very early in the disease, and is extremely common in all cases of chronic suppuration—that is in those which have lasted for over two years. Of the walls of the tympanum, the attic and the margins of the aditus, or opening to the antrum, are the parts most often attacked. Bone disease should be suspected when polypi or granulations recur obstinately after removal. Caries is practically always present in cases of attic suppuration, with a perforation of Shrapnell's membrane or high up in the postero-superior quadrant of the drum. In cases of attic suppuration a cure may be sometimes obtained by intra-tympanic syringing with a very fine bent cannula passed into the attic through the perforation. A mild lotion should be used, and may be followed by the injection of a few drops of rectified spirit. If this is unsuccessful, and, indeed, in most cases where caries is present, the only alternative to the radical post-aural operation is ossiculectomy. This expression means the removal of the malleus and incus, but the operation also includes the curetting away of granulations and any carious patches on the tympanic walls, and usually also nowadays the removal of the outer wall of the attic so as to throw this cavity into free communication with the meatus. This operation is indicated in cases of attic suppuration, which conservative measures, including intra-tympanic syringing, have failed to cure, and in cases where caries of the ossicles can be demonstrated, but there are no signs of serious involvement of the mastoid. Sometimes, where the complete mastoid operation appears to be indicated, ossiculectomy by improving the drainage from the antrum may effect a cure, and even when it falls short of this it diminishes the future danger of serious complications. It may, therefore, be employed, in such cases as show no signs of severe complications, as an occasional substitute for the larger operation, provided that it be well understood that the latter may have to be performed later if the smaller procedure fails to effect a cure. There is considerable difference of opinion as to the advisability of performing ossiculectomy before proceeding to the post-aural operation in cases of persistent chronic suppuration without severe symptoms.

The principal indications for the complete mastoid operation, as generally recognised, are as follows. Acute inflammation of the mastoid occurring in the course of a chronic suppuration; tuberculosis of the middle ear, if the general health permits; intracranial complications, the disease being followed into the part of the brain affected from its origin in the ear; facial paralysis occurring in the course of a chronic aural suppuration; fistulæ through the skin, either in the meatus or externally; cholesteatomata; middle-ear suppuration, complicated by headache, sickness, or giddiness, in spite of cleansing and conservative treatment; frequently recurring polypi, though in this case ossiculectomy may be given a trial; persistence of discharge, especially if fœtid, in spite of proper conservative treatment. In cases of otorrhœa, with no special symptoms, which have persisted for a year or two in spite of careful treatment, including any necessary operation on the nose or naso-pharynx, it is a somewhat difficult question to decide whether operation should be advised. When the perforation is large, and not high up, the discharge free from odour, and the patient within reach of skilled help, such cases may be left without great danger, if the risk be fully explained to the patient, and the importance of constant care of the ear impressed on him; but in all other circumstances operation should be advised when otorrhœa has continued for over two years in spite of proper care, and, indeed, it is safer to recommend it in every case of this kind.

